# Asthma-susceptibility variants identified using probands in case-control and family-based analyses

**DOI:** 10.1186/1471-2350-11-122

**Published:** 2010-08-10

**Authors:** Blanca E Himes, Jessica Lasky-Su, Ann C Wu, Jemma B Wilk, Gary M Hunninghake, Barbara Klanderman, Amy J Murphy, Ross Lazarus, Manuel E Soto-Quiros, Lydiana Avila, Juan C Celedón, Christoph Lange, George T O'Connor, Benjamin A Raby, Edwin K Silverman, Scott T Weiss

**Affiliations:** 1 Harvard-MIT Division of Health Sciences and Technology, Cambridge, MA, USA; 2 Channing Laboratory, Brigham and Women's Hospital and Harvard Medical School, Boston, MA, USA; 3 Children's Hospital Informatics Program, Boston, MA, USA; 4 Partners Center for Personalized Genetic Medicine, Boston, MA, USA; 5 Department of Population Medicine, Harvard Medical School and Harvard Pilgrim Health Care Institute, Boston, MA, USA; 6 Department of General Pediatrics, Children's Hospital, Boston, MA, USA; 7 Department of Medicine, Boston University School of Medicine, Boston, MA, USA; 8 Division of Pediatric Pulmonology, Hospital Nacional de Niños, San José, Costa Rica

## Abstract

**Background:**

Asthma is a chronic respiratory disease whose genetic basis has been explored for over two decades, most recently via genome-wide association studies. We sought to find asthma-susceptibility variants by using probands from a single population in both family-based and case-control association designs.

**Methods:**

We used probands from the Childhood Asthma Management Program (CAMP) in two primary genome-wide association study designs: (1) probands were combined with publicly available population controls in a case-control design, and (2) probands and their parents were used in a family-based design. We followed a two-stage replication process utilizing three independent populations to validate our primary findings.

**Results:**

We found that single nucleotide polymorphisms with similar case-control and family-based association results were more likely to replicate in the independent populations, than those with the smallest p-values in either the case-control or family-based design alone. The single nucleotide polymorphism that showed the strongest evidence for association to asthma was rs17572584, which replicated in 2/3 independent populations with an overall p-value among replication populations of 3.5E-05. This variant is near a gene that encodes an enzyme that has been implicated to act coordinately with modulators of Th2 cell differentiation and is expressed in human lung.

**Conclusions:**

Our results suggest that using probands from family-based studies in case-control designs, and combining results of both family-based and case-control approaches, may be a way to augment our ability to find SNPs associated with asthma and other complex diseases.

## Background

Asthma [MIM 600807] is a chronic respiratory disease that affects over 20 million Americans and 300 million people worldwide [1,2]. The genetic basis of asthma has been explored for over two decades in candidate gene association, where more than 40 genes have been associated with asthma and replicated in at least one independent population [3,4]. Recently, genome-wide association (GWA) studies of asthma have found that variants in or near several genes, including *ORMDL3 *[MIM 610075] [5-12], *CHI3L1 *[MIM 601525] [13], *TLE4 *[MIM 605132] [14], *PDE4D *[MIM 600129] [15], *DENND1B *[MIM 613292] [16], *RAD50-IL13 *[MIM 604040, 147683] [17], and the *HLA-DR/DQ *region on chromosome 6p21.3 [17], contribute to the risk of asthma.

The primary findings of the *PDE4D *GWA study were obtained in a case-control design consisting of children from an asthma clinical trial and publicly available population controls. Although we used a case-control design, genetic studies of these asthmatic children were originally intended to be part of a family-based design, as DNA was collected from children and their parents. We chose the case-control design in an effort to increase the power to detect genetic associations. Consistent with the approach taken in most GWA studies, we selected the variants with the lowest p-values in our primary population for replication in independent populations. Here, we extensively compare the GWA results of the case-control and family-based designs using probands from the same population, and we attempt to replicate the initial association findings of many SNPs with nominally significant p-values in a two-stage process. Our results help to determine whether the case-control design, the family-based design, or a combination of results from both designs, is more powerful to identify asthma-susceptibility variants.

## Methods

### Subjects

Our primary population is composed of 422 non-Hispanic white subjects from the Childhood Asthma Management Program (CAMP), a clinical trial that followed 1,041 asthmatic children for four years and nearly 80% of the original participants for 12 years [18]. CAMP participants and their parents provided DNA for family-based genetic studies. Additionally, CAMP probands were used in a case-control design by matching them with 1,533 white population controls that are publicly available through the Illumina iControlDB resource (http://www.illumina.com/science/icontroldb.ilmn) [15].

### Genotyping and Quality Control

Genome-wide SNP genotyping for 422 Caucasian CAMP subjects, their families, and iControlDB controls was performed on Illumina's HumanHap550 Genotyping BeadChip (Illumina, Inc., San Diego, CA). Details of the quality control (QC) criteria used to screen the genome-wide SNP data have been provided previously [15]. Briefly, of the 422 CAMP subjects who were genotyped, 403 had genotyping completion rates greater than 95% and were used in subsequent analyses. SNPs were excluded for having low clustering scores (n = 6,257), flanking sequences that did not map to a unique position on the HG17 reference genome (n = 1,329), having 5 or more Mendel errors (n = 2,445), or being monomorphic (n = 3790). For the family-based analysis, hereafter referred to as CAMP Trio study, 534,290 SNPs in 403 probands and their parents passed QC filters. For the case-control analysis, additional QC filters were used. Subjects were excluded for being siblings of other subjects (23 cases) or showing evidence of identity by descent (IBD) (57 controls) or sex discordance (3 controls). SNPs were excluded if they were missing in more than 5% of subjects (n = 3,837), had minor allele frequency (MAF) less than 1% (n = 17,088), had Hardy-Weinberg equilibrium p-values among controls < 0.001 (n = 2,046), or had a significantly different (p-value < 1E-05) missing rate in cases and controls (n = 6,642). After these QC filters, 518,230 SNPs remained. Genetic matching (GEM) [19] was used to control for population stratification, and as a result of implementing this procedure, 21 cases and 687 controls were dropped. The remaining 1,205 subjects (359 cases, 846 controls) had a genomic inflation factor of 1.03, demonstrating minimal population stratification. After re-enforcing a SNP MAF threshold of greater than 1% in the remaining subjects, 516,617 SNPs remained for the analysis of CAMP cases and Illumina controls, which will hereafter be referred to as the CAMP/Illumina study.

### Statistical Analysis

**Figure 1:**
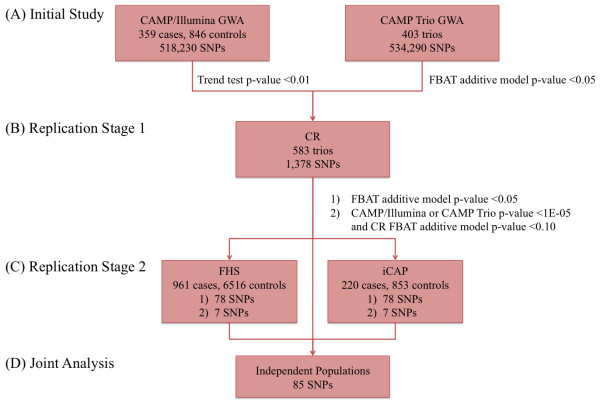
**Study Design**. (A) The initial study population consisted of CAMP probands used in (1) a case-control design composed of 359 CAMP cases and 846 Illumina controls and (2) a family-based design composed of 403 trios. Genome-wide association of individual SNPs to asthma status was assessed in each of these designs. SNPs with a case-control Cochran-Armitage trend test p-value < 0.01 and a family-based PBAT additive model p-value < 0.05 were selected for replication analysis. (B) The first replication stage, carried out in CR, measured the association of 1378 SNPs with asthma. Those SNPs with either (1) a CR PBAT additive model p-value < 0.05 or (2) a CR PBAT additive model p-value < 0.10 and a p-value < 1E-05 from the designs in (A) were selected for the next replication stage. (C) The second replication stage was carried out in two additional independent populations, FHS and iCAP. (D) Joint association analysis for 85 SNPs with data in the three independent populations was performed.

Figure 1 is an overview of our study design. First, we performed CAMP/Illumina and CAMP Trio GWA analyses [Figure 1A]. SNPs for further consideration were selected by choosing nominal p-value cutoffs in both the case-control and family-based analyses. Case-control associations were measured in PLINK [20] using Cochran-Armitage trend tests. Family-based association statistics for 403 CAMP trios, assuming an additive model of inheritance, were calculated using PBAT version 3.6 [21]. Those SNPs with CAMP/Illumina Cochran-Armitage trend test p-values < 0.01 and FBAT additive model p-values < 0.05 were selected for replication in an initial independent population [Figure 1B]. Further replication of association results was attempted in two additional independent populations [Figure 1C]. This two-staged replication approach was taken so that a larger number of SNPs could be genotyped in the initial replication population, which was most similar to CAMP as it was also composed of children who were carefully ascertained for asthma studies. Subsequently, a smaller number of SNPs that successfully replicated in the initial replication population could be tested in two additional populations. Joint evidence for association across replication populations was measured by combining p-values using the Liptak method [22] [Figure 1D]. In combining p-values, all hypothesis tests in replication populations had one-sided alternatives (based on the direction of the association in the testing population) so that SNPs with association tests in opposite directions would not produce inappropriately small p-values. Effect estimates were calculated using allelic odds ratios (ORs) for case-control data. To evaluate directionality of effect in trios, transmitted to untransmitted ratios (T:U) were calculated in Haploview. Power calculations for "TDT with discrete traits" and "case-control with discrete traits" designs were performed using the Genetic Power Calculator by Purcell S, *et al *[23] with high risk and marker allele frequencies of 0.10/0.40, prevalence of 0.10, D-prime of 1, assuming use of unselected controls for the case-control statistics, and with default error rates (alpha = 0.05, power = 0.80).

### Replication Studies

(1) CR. This cohort consists of 592 probands from the Genetics of Asthma in Costa Rica Study (CR), which is comprised of Costa Rican schoolchildren with asthma and their parents [24,25]. Children had a high probability of having at least six great-grandparents born in the Central Valley of Costa Rica and were defined as having asthma if they had a doctor's diagnosis of asthma and at least two respiratory symptoms or asthma attacks in the year prior to enrollment in the study. Most genotype data to replicate CAMP/Illumina findings was obtained with an Illumina 1536 GoldenGate assay. Of the 1536 SNPs attempted, 1375 met the following quality thresholds for analysis, after removal of 9 failed subjects: 1) completion rate > 95%, 2) three or fewer Mendelian inconsistencies, 3) zero discordance among replicate samples, and 4) MAF > 0. Data for 2 SNPs (rs261119, rs2777899) were obtained with the SEQUENOM MassARRAY system (Sequenom, Inc., San Diego, CA), which utilizes iPlex chemistry. Data for one SNP (rs11778371) was obtained with Taqman real-time PCR with an ABI Prism 7900 machine (Applied Biosystems, Foster City, CA). Standard PCR conditions, as recommended by the manufacturer, were used. Overall, 583 trios had good genotype data available for 1378 SNPs. Family-based association statistics for asthma affection status under an additive model were calculated using Golden Helix PBAT version 6.4.0 [21]. T:U of alleles were calculated in Haploview [26].

(2) FHS. The Framingham Heart Study (FHS) is a family-based study that conducted clinical examinations, including spirometry and collection of smoking history data, on three generations of white adults of European descent, and research participants provided DNA samples that have recently been genotyped for genome-wide association studies [27,28]. Asthma was classified based on self-report of physician diagnosis, and according to this definition, there were 961 cases and 6,516 controls. In FHS subjects, genotyping was performed using the Affymetrix GeneChip Human Mapping 500 K Array Set and an additional Affymetrix 50 K Array (HuGeneFocused50K). Because data from these assays did not include that of some associated SNPs that passed the replication stage in CR, those genotypes were inferred using imputation with the Markov Chain Haplotyping software (MaCH) [29]. The ratio of the empirically observed dosage variance to the expected (binomial) dosage variance for these imputed SNPs was greater than 0.9, indicating good quality of imputation. Association to asthma was measured using logistic regression models with robust variance estimated via generalized estimating equations with each pedigree as a cluster, while adjusting for age, former smoking, current smoking, pack-years, sex, BMI, and membership in one of the three recruited generations. The genomic inflation factor for the imputed genome-wide results was 1.048, indicating minimal population stratification.

(3) iCAP. The i2b2 Crimson Asthma Project (iCAP) consists of Partners Healthcare System, Inc. (Boston, MA) patients who were selected based on extracted de-identified electronic medical record (EMR) data and whose DNA was obtained via discarded clinical samples. Specifically, tools developed by the National Center for Biomedical Computing entitled "Informatics for Integrating Biology to the Bedside" (i2b2, http://www.i2b2.org) have facilitated extraction of de-identified demographic and clinical information from EMRs of patients. Using i2b2 resources, a large set of asthmatic and non-asthmatic Partners Healthcare patients has been identified on the basis of International Classification of Diseases, Ninth Revision (ICD-9) codes for asthma (i.e. those beginning with 493) [30,31]. In order to conduct genomic studies of these patients, clinical samples that are routinely collected at healthcare visits were obtained via the Crimson Project (http://www.crimsonproject.org), which identifies discarded Partners Healthcare clinical samples that are ordered for routine clinical tests, and prospectively collects the samples that have been requested by an approved study. For this study, to further ensure that cases truly had asthma, medication history extracted from EMR records was utilized. Cases (n = 220) were defined as those patients whose EMRs contained an asthma ICD-9 code and whose medication history included usage of at least one beta-agonist or inhaled corticosteroid. Controls (n = 853) were selected as those patients who had been seen in the three years prior to blood collection in at least one of over 850 outpatient clinics but did not have any asthma ICD-9 codes. The gender composition of cases (19.5% male, 80.5% female) and controls (15.8% male, 84.2% female) was not statistically different (Fisher's exact p-value 0.19) although there is a high prevalence of female subjects overall due to a large portion of patients being recruited at Brigham and Women's Hospital, which has a high proportion of female patients. There was no significant difference in age between cases (mean = 28.8 years [SD 6.0, range = 4 to 35]) and controls (mean = 30.0 years [SD 4.1, range = 18 to 35]) at the time of DNA collection (Wilcoxon rank sum p-value = 0.082). Genotyping of the SNPs of interest was carried out with an Illumina GoldenGate assay. Markers were analyzed if they met the same quality standards as described for Costa Rica genotyping, with the exception of Mendelian checks, which were not applicable. Genotyped SNPs included those for replication of association to asthma as well as two panels of SNPs to measure population stratification: (1) a set of intergenic SNPs selected randomly throughout the genome [32], and (2) a set of ancestry informative markers (AIMs) [33]. The random panel of 187 SNPs had an association χ^2 ^_187 df _= 168.7, corresponding to a p-value of 0.83. The set of 248 AIM SNPs was used to compute principal components describing variation in iCAP subject data using EIGENSTRAT [34]. The principal components were used to obtain EIGENSTRAT-corrected association statistics for replication SNPs. The r^2 ^between corrected and uncorrected association statistics was 0.95. Thus, no significant evidence of population stratification was found by two methods.

## Results

Probands from CAMP were used to measure association of SNPs to asthma using both a case-control and a family-based GWA study design. The top SNPs according to each design are shown in Table 1. In the case-control design (i.e. CAMP/Illumina), which utilized publicly available population controls, 516,617 SNPs in 1,205 subjects (359 cases, 846 controls) were evaluated for association using the Cochran-Armitage trend test, and 15 SNPs had p-values < 1E-05. In the family-based GWA (i.e. CAMP Trio study), 534,290 SNPs in 403 trios were ranked according to PBAT additive model p-values, and 13 SNPs had p-values less than a nominally significant level of 1E-05. There was no overlap among the top (i.e. those with p-value < 1E-05) CAMP/Illumina and CAMP Trio SNPs.

**Table 1:** Top-ranked SNPs in each Initial Study (CAMP/Illumina or CAMP Trio p-value < 1E-05)

			Rank		Discovery P-values			Stage 1	Stage 2		
						
SNP	CHR	BP	CAMP/Illumina	CAMP Trio	CAMP/Illumina	CAMP Trio	Replication Attempted?	CR	FHS	CAP	Liptak P-value*
Top CAMP/Illumina											

rs2548659	5	59419643	1	3122	2.07E-07	5.9E-03	yes	0.064	NA	0.15	0.035
rs1588265	5	59405551	2	5000	5.11E-07	9.4E-03	reported in [15]	-	-	-	-
rs983280	5	59480894	3	10253	5.71E-07	0.019	yes	0.094	5.7E-03	NA	3.3E-03
rs11778371	8	27375822	4	2947	7.92E-07	5.6E-03	yes	0.29	-	-	-
rs1544791	5	59474839	5	10852	1.16E-06	0.020	yes/reported in [15]	0.15	-	-	-
rs684909	11	35843495	6	7849	3.12E-06	0.015	yes	0.99	-	-	-
rs12725071	1	105022488	7	306415	4.52E-06	0.57	no	-	-	-	-
rs17219773	4	61926823	8	11376	4.56E-06	0.021	yes	0.83	-	-	-
rs12930287	16	64495907	9	228338	4.80E-06	0.42	no	-	-	-	-
rs11751990	6	130829469	10	19852	4.97E-06	0.037	yes	0.018	0.81	0.43	0.21
rs2761647	23	95159314	11	NA	5.83E-06	NA	no	-	-	-	-
rs12724129	1	11880226	12	43354	6.83E-06	0.082	no	-	-	-	-
rs7765374	6	87001318	13	8269	7.34E-06	0.015	yes	0.18	-	-	-
rs2910830	5	59502960	14	16890	7.79E-06	0.032	yes	0.069	0.061	NA	0.016
rs9318942	13	82928515	15	34248	8.72E-06	0.065	no	-	-	-	-

Top CAMP Trio											

rs1288548	4	186536979	4955	1	8.6E-03	8.7E-07	yes	0.24	-	-	-
rs261137	5	4414120	1068	2	1.8E-03	1.2E-06	yes	0.053	0.26	0.27	0.049
rs12734338	1	200736346	NA	3	NA	1.7E-06	no	-	-	-	-
rs1048329	4	186536752	1637	4	2.8E-03	3.1E-06	yes	0.29	-	-	-
rs261159	5	4396987	212	5	3.3E-04	3.4E-06	yes	0.086	0.29	0.27	0.072
rs12247820	10	53272196	558	6	9.0E-04	4.2E-06	yes	0.74	-	-	-
rs12743401	1	200743271	NA	7	NA	4.2E-06	no	-	-	-	-
rs261119	5	4418468	4995	8	8.7E-03	4.2E-06	yes	0.44	-	-	-
rs1039603	5	4345616	309	9	4.8E-04	5.0E-06	yes	0.063	0.32	NA	0.079
rs2777899	17	55187173	828	10	1.4E-03	6.1E-06	yes	0.34	-	-	-
rs261125	5	4422887	3764	11	6.5E-03	6.6E-06	yes	0.62	-	-	-
rs13267437	8	4183474	20383	12	0.037	9.5E-06	no	-	-	-	-
rs9463425	6	48620994	79984	13	0.15	9.7E-06	no	-	-	-	-

*In all available independent populations

**Figure 2:**
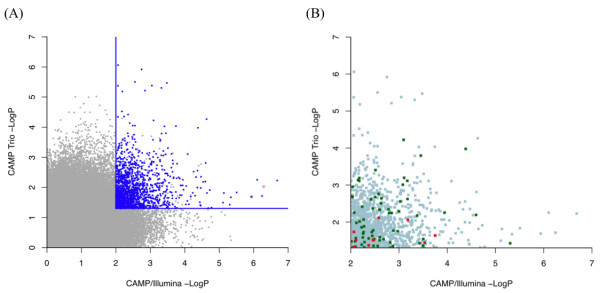
**Association Results**. (A) Plot of CAMP Trio vs. CAMP/Illumina GWA results. SNPs selected for replication analysis have family-based PBAT additive model p-values < 0.05 and case-control Cochran-Armitage trend test p-values < 0.01. The subset of these SNPs that was genotyped in CR is shown in blue. Points with a pink background are SNPs whose association in several populations was reported previously [15]. (B) Plot of CAMP Trio vs. CAMP/Illumina GWA results for the subset of SNPs that was successfully genotyped in CR. Shown in green are those SNPs with CR p-values < 0.05. Shown in red are those SNPs that have CR p-values < 0.05 and either a FHS or iCAP p-value < 0.05.

Under the assumption that consistency of results in CAMP/Illumina and CAMP Trio increased the likelihood of an association being true, we proceeded to replicate those SNPs with CAMP/Illumina p-value < 0.01 and CAMP Trio p-value < 0.05 [Figure 2A]. Such thresholds were selected to have enough SNPs to fill a genotyping assay for 1536 SNPs, and the lower p-value threshold for CAMP/Illumina was chosen because of the increased power that CAMP/Illumina had to detect associations relative to CAMP Trio. Of 5604 SNPs with CAMP/Illumina p-values < 0.01, 1726 SNPs had CAMP Trio p-values < 0.05. Among this set of 1726 SNPs were some of the top SNPs according to the individual primary analyses: 9 CAMP Trio SNPs with p-values < 1E-05, and 9 CAMP/Illumina SNPs with p-values < 1E-05 [Table 1]. One (rs1588265) of the top 10 CAMP/Illumina SNPs that had CAMP Trio p-value < 0.05 was not genotyped because it was replicated previously in independent populations, including CR and FHS, and is in very tight linkage disequilibrium with three of the 9/10 other replicated SNPs [15].

For replication Stage 1, a subset of 1378 of the 1726 SNPs was successfully genotyped in CR, an independent population of children with asthma [Figure 2A]. Because of our selected genotyping platform, an Illumina 1536 GoldenGate assay, we had to constrain the initial set of 1726 SNPs to 1536. Most SNPs were excluded based on LD: we attempted to capture all regions of association by selecting at least one SNP within sets of SNPs that were in strong (pairwise r^2 ^> 0.80) LD, but we excluded remaining SNPs within such sets. However, some SNPs were excluded because they had low GoldenGate assay design scores while others were excluded because they failed the GoldenGate assay. Three of the SNPs (rs261119, rs2777899, rs11778371) that failed the GoldenGate assay were genotyped by other methods in Stage 1 because they were among the top-ranked SNPs in the family-based and case-control designs, and we felt that their results were essential to compare the replication of SNPs selected based on the different study designs. Of the 1378 SNPs that were successfully genotyped in Stage 1, 78 had 1-sided p-values < 0.05 with CR effects in the same direction as CAMP [Figure 2B, Table 2, Additional file 1, Table S1]. Only one (rs11751990) of these 78 SNPs was among the top-ranked SNPs from the individual studies shown in Table 1.

**Table 2:** SNPs that replicated in 2 of 3 independent populations

			Discovery P-values		Rank		Replication P-values					
						
							Stage 1	Stage 2				
										
SNP	CHR	BP	CAMP/Illumina	CAMP Trio	CAMP/Illumina	CAMP Trio	CR	FHS	iCAP	Liptak P-value	Nearest RefSeq Gene(s)	Distance From Gene
rs17572584	3	144370733	4.3E-03	0.043	2539	22513	1.4E-03	0.011	0.052	3.5E-05	CHST2	46233
rs10489341	1	207998744	8.6E-03	0.047	4946	25122	1.9E-03	0.023	0.056	9.2E-05	TRAF3I	In gene
rs4653637	1	223690639	2.9E-04	0.036	189	19134	0.012	0.23	6.0E-03	7.6E-04	LBR	In gene
rs530914	11	95271804	7.8E-03	0.031	4525	16196	8.9E-03	0.14	0.035	1.2E-03	MTMR2	In gene
rs10816789	9	111032330	3.5E-03	0.030	2082	16121	0.012	6.9E-03	0.29	1.2E-03	EPB41L	In gene
rs247052	16	56524719	1.8E-04	0.023	128	12253	0.036	3.3E-03	0.27	1.5E-03	CNGB1	In gene
rs1534837	14	79452778	6.5E-04	8.8E-03	416	4617	0.047	0.059	0.037	1.9E-03	NRXN3	52265
rs714679	11	44610240	2.6E-03	7.7E-03	1542	4053	0.032	0.15	0.028	2.9E-03	CD82	12351
rs11947034	4	35448626	8.6E-03	0.018	4963	9830	0.013	0.43	0.020	4.8E-03	CENTD1	295390
rs1152490	14	55865987	3.4E-03	0.031	2029	16236	0.045	0.039	0.21	6.6E-03	PELI2	28204

For replication Stage 2, 85 SNPs were evaluated in two additional independent populations, FHS and iCAP. These SNPs included the 78 SNPs with CR p-values < 0.05 and an additional 7 SNPs with CR p-values < 0.10 that were part of the top CAMP/Illumina and CAMP Trio SNPs. We found that 10 SNPs had a p-value < 0.05 in FHS or iCAP and had the same direction of effect across all populations [Tables 2, 3 and 4]. Overall Liptak p-values in the three independent populations revealed that one SNP passes a multiple comparisons correction threshold of 3.6E-05 (= 0.05/1378), corresponding to the number of SNPs genotyped in CR.

**Table 3:** Minor Allele Frequencies of SNPs that replicated in 2 of 3 independent populations

		CAMP/Illumina		CAMP Trio		CR		FHS		iCAP	
**SNP**	**Minor Allele**	**Affected**	**Unaffected**	**Affected**	**Unaffected**	**Affected**	**Unaffected**	**Affected**	**Unaffected**	**Affected**	**Unaffected**

rs17572584	T	0.078	0.12	0.085	0.10	0.057	0.074	0.083	0.10	0.073	0.10
rs10489341	T	0.036	0.018	0.036	0.027	0.058	0.046	0.028	0.022	0.050	0.033
rs4653637	G	0.40	0.32	0.39	0.37	0.34	0.32	0.32	0.31	0.39	0.32
rs530914	C	0.54	0.48	0.52	0.50	0.54	0.47	0.51	0.49	0.51	0.46
rs10816789	T	0.19	0.14	0.19	0.18	0.14	0.13	0.18	0.16	0.15	0.14
rs247052	T	0.26	0.34	0.26	0.28	0.33	0.35	0.28	0.31	0.29	0.30
rs1534837	A	0.042	0.079	0.046	0.057	0.011	0.014	0.065	0.074	0.055	0.080
rs714679	G	0.39	0.46	0.41	0.43	0.33	0.34	0.42	0.43	0.40	0.45
rs11947034	C	0.033	0.060	0.041	0.052	0.054	0.063	0.061	0.062	0.050	0.079
rs1152490	C	0.18	0.24	0.18	0.21	0.20	0.21	0.21	0.23	0.21	0.23

**Table 4:** Association directions of SNPs that replicated in 2 of 3 independent populations

SNP	CAMP/Illumina	CAMP Trio	CR	FHS	iCAP
rs17572584	0.63 (0.47-0.87)	0.71	0.61	0.82 (0.75-0.89)	0.72 (0.49-1.07)
rs10489341	2.01 (1.19-3.42)	1.87	1.83	1.33 (1.15-1.53)	1.52 (0.92-2.52)
rs4653637	1.40 (1.17-1.67)	1.25	1.24	1.04 (0.98-1.10)	1.32 (1.06-1.64)
rs530914	1.26 (1.06-1.51)	1.24	1.23	1.06 (1.00-1.12)	1.21 (0.98-1.50)
rs10816789	1.43 (1.13-1.80)	1.36	1.26	1.20 (1.12-1.30)	1.09 (0.81-1.46)
rs247052	0.69 (0.57-0.84)	0.77	0.85	0.84 (0.79-0.90)	0.93 (0.74-1.17)
rs1534837	0.51 (0.34-0.76)	0.57	0.53	0.85 (0.77-0.94)	0.67 (0.43-1.04)
rs714679	0.76 (0.63-0.91)	0.75	0.86	0.94 (0.88-1.00)	0.81 (0.66-1.01)
rs11947034	0.54 (0.35-0.86)	0.55	0.67	0.98 (0.89-1.08)	0.62 (0.39-0.98)
rs1152490	0.72 (0.57-0.90)	0.75	0.85	0.89 (0.83-0.95)	0.90 (0.70-1.16)

CAMP/Illumina, FHS, and iCAP columns contain ORs (95% confidence intervals). CAMP Trio and CR columns contain T:Us.

Three additional SNPs on chromosome 17q21 near *ORMDL3 *were evaluated in all populations using previously published results for CAMP/Illumina [15] and CAMP and CR trios [12], and novel results for CAMP Trio, FHS and iCAP [Table 5].

**Table 5:** Replication of 3 Chromosome 17 SNPs near ORMDL3 initially reported by Moffat et al. [5]

SNP	BP	CAMP/Illumina	CAMP Trio	**CAMP trios Reported in **[12]	**CR Reported in **[12]	FHS	iCAP
rs9303277	35229995	0.011	0.041	4.0E-03	1.5E-05	8.6E-03	3.3E-03
rs8067378	35304874	7.0E-03	0.10	7.0E-03	8.1E-06	0.019	8.4E-03
rs7216389	35323475	1.7E-03	0.15	0.13	9.3E-06	0.023	0.040

Associations were in the same direction (data not shown). "CAMP Trio" refers to GWA results corresponding to the 403 trios reported in the current study. "CAMP trios Reported in [12]" refers to results from a previous candidate gene study corresponding to 444 trios.

## Discussion

The CAMP population was designed for, and has often been used in, family-based candidate gene association studies [35-37]. More recently, GWA data have been acquired for a cohort of 403 Caucasian probands and their parents. This cohort has low statistical power to detect associations corresponding to the expected small (i.e. 1.1-1.3) effect sizes that have been observed to underlie many complex traits, including asthma [38]. We sought to increase statistical power by using the CAMP probands as cases in a case-control design, where controls were obtained from Illumina's iControlDB resource. Although we increased statistical power with the case-control design, which is composed of 359 cases and 846 controls, this design also remains underpowered to detect associations of small effect sizes. For example, for a SNP such as rs17572584, which has an MAF near 0.10 and an effect size of 1.6, the power to detect its association to asthma via a TDT in CAMP Trio is 0.37, while the power in CAMP/Illumina is 0.45. For a SNP such as rs4653637, which has an MAF near 0.40 and an effect size of 1.4, the power in CAMP Trio is 0.39, while the power in CAMP/Illumina is 0.47. Thus, despite a significant increase in power, neither design individually has a large enough number of subjects to make it adequate to detect most of the small effect sizes that are expected for asthma. In this work, we attempted to find out whether a combination of the results of the two designs would be helpful to find SNPs that replicated in independent populations, and hence, were likely to be truly associated with asthma.

The top SNPs according to the results for individual designs did not overlap [Figure 2], and only 1/18 of the top SNPs that were genotyped for replication Stage 1 had a p-value < 0.05 [Table 1]. Because of the low p-values in either the CAMP/Illumina or CAMP Trio designs, we were more lenient with the replication threshold for these SNPs to be analyzed for Stage 2, and proceeded to consider the SNPs with p-value < 0.10 in CR. Based on the overall results in Table 1, only the three CAMP/Illumina SNPs on Chromosome 5 (Table 1, CAMP/Illumina Rank 1, 3, 14) show evidence of association to asthma. These three SNPs and the two others near them on Chromosome 5 (Table 1, CAMP/Illumina Rank 1-3, 5, 14) are near and within sequences of the *PDE4D *gene as we reported previously [15]. In this previous work, we attempted to replicate the top CAMP/Illumina findings in independent populations. We found that in 2/7 independent populations our results replicated with p < 0.05 and that in 5/7 the results did not replicate but had consistent effect directions with CAMP/Illumina, providing overall p-values in the independent populations that supported the association of *PDE4D *variants with asthma. These previous findings would have been more difficult to identify based on our current replication strategy because the CR population did not convincingly replicate the *PDE4D *associations. Thus, having the first stage be a filtering stage limits our ability to identify some of the SNPs that may be truly associated with asthma because they may not replicate in an initial independent population but would in other independent populations. This limitation may be overcome in future studies that pool GWA results across multiple populations.

Most of the SNPs that passed replication Stage 1 were distributed closer to the nominal p-value thresholds than to the low p-value extremes of either CAMP/Illumina or CAMP Trio [Figure 2B in green]. The SNPs that passed replication Stage 2 were even closer to the nominal p-value thresholds than those of replication Stage 1 [Figure 2B in red]. Thus, SNPs with consistent results in the two initial study designs were more likely to replicate in independent populations than those with extreme p-values in either individual design. Tables 2, 3 and 4 detail the characteristics of the SNPs that passed replication Stage 2 (i.e. had p-value < 0.05 in CR and either FHS or iCAP, with consistent effect direction across all populations), and hence, are most likely to be truly associated with asthma. Most of these SNPs are in or near genes [Table 2], making them more likely to have biological relevance than SNPs that are often found to be associated with asthma and are in gene deserts. The top SNP (rs17572584) in Table 2 has an overall p-value across the independent populations that is significant after multiple comparisons corrections for the number of SNPs that were genotyped in Stage 1 (p-value = 0.048). If the results for this SNP in the independent populations are combined with those of the primary CAMP studies, then the overall p-value would be 9.7E-07 with CAMP/Illumina and 8.3E-06 with CAMP Trio. The rs17572584 SNP is downstream of the carbohydrate (N-acetylglucosamine-6-O) sulfotransferase 2 gene (*CHST2 *[MIM 603798]), which encodes a sulfotransferase for 6-sulfated glycan synthesis. Transcription of this gene has been found to be coordinated with that of NF-kappaB and GATA-3, both of which are involved in Th2 cell differentiation [39]. Because Th2 cells are prominent in asthma, especially allergic asthma, genes such as *CHST2 *that modulate Th2 cell differentiation are likely to play a role in asthma susceptibility. Further, the *CHST2 *protein product has been shown to be expressed in the human lung [40]. Even though *CHST2 *is a plausible candidate gene for asthma, rs17572584 is located downstream of this gene, and hence, further study is needed to find out whether the association we measured is stronger in SNPs located nearer or within the gene across multiple populations. However, identification of this association using either CAMP/Illumina or CAMP Trio alone would have been difficult since this SNP ranked 2,539 and 22,513 in these primary studies, respectively. The fact that our strongest associations were located in regions that were not at the lowest p-value extremes, but were at nominally significant levels, reflects the potential of small populations to contain useful genetic associations that can be found by increases in power. As stated previously, this power limitation may be overcome in future studies that pool GWA results across multiple populations.

Currently, among regions reported to be associated with asthma, the one on chromosome 17q21 near *ORMDL3 *[MIM 610075] [5-12] has been the most consistently replicated. We previously reported [15] that nine CAMP/Illumina SNPs support the original association findings by Moffatt et al. in this region [5], by having p-values < 0.05. Of these nine SNPs, only one (rs9303277) has a CAMP Trio p-value < 0.05. However, this SNP was excluded from our Replication Stage 1 because its CAMP/Illumina p-value was equal to 0.011 (i.e. was greater than our 0.010 cutoff). In a separate study of association in the 17q21 region, rs9303277 was reported to have a p-value = 4.0E-03 in CAMP trios and = 1.5E-05 in CR trios [12]. The lower CAMP p-value reported in [12] was obtained using TaqMan SNP Genotyping Assays (Applied Biosystems) data corresponding to 444 white trios, and not the GWA data corresponding to 403 trios reported in the current study. The difference in CAMP p-values as a result of using a slightly increased number of trios and different genotyping platforms is another reflection of the low power of CAMP Trio, and suggests that additional SNPs with more modest p-values than those considered by our replication strategy are truly associated with asthma. Because the 17q21 region near *ORMDL3 *has been consistently replicated in so many studies, including CAMP and CR, we evaluated the association for three of the SNPs originally reported by Moffat, et al. in iCAP and FHS. As Table 5 shows, these three SNPs replicated with nominal p-values < 0.05 in these two populations, growing the total number of independent populations replicating this region's results to over 12. Additionally, the replication of the chromosome 17q21 results in FHS and iCAP helps validate the use of these populations, which were not ascertained to have asthma using as strict criteria as CAMP and CR, in asthma association studies.

In previous work that developed a method to identify reproducible associations in small affected trio-based cohorts, two CAMP Trio SNPs were found to reach genome-wide significance according to the developed criteria [41]. These SNPs (rs10863712 and rs1294497) ranked first and second according to an initial power screen and had FBAT p-values that passed the corresponding genome-wide significance thresholds (i.e. p-value < 0.005). The CAMP/Illumina p-values (ranks) for these two SNPs were 0.28 (149891) and 0.049 (27336) for rs10863712 and rs1294497, respectively. The CAMP Trio p-values (ranks) for these two SNPs were 0.0032 (1684) and 0.0048 (2547) for rs10863712 and rs1294497, respectively. Thus, neither of these SNPs met the criteria for replication Stage 1 and neither was tested for replication in our independent cohorts. Future attempts to replicate the top SNPs according to the power-based screening methodology would be helpful to compare its utility with that of the current approach of combining case-control and family-based results.

Our study has limitations similar to those of many other GWA studies. In addition to having low power to detect associations and some missing genotypic data for all of our populations, there were differences in the way our populations were ascertained to have asthma. CAMP and CR are the most similar populations, as they are composed of children with asthma who were carefully ascertained for asthma studies. However, the two populations are ethnically different and have markedly different environmental exposures. The FHS population was not initially gathered for asthma research, but asthma status has been assessed for its participants based on self-reported doctor's diagnosis. The iCAP cohort was selected based on data, including ICD-9 codes for asthma and medication history, extracted from de-identified EMRs. The diversity of our populations increases the certitude that the SNPs that are in Table 2 are general asthma variants, rather than variants that are associated to asthma sub-phenotypes that characterize each of the populations. Any errors in the assignment of cases and controls would decrease our ability to find associations, and thus, does not increase the likelihood that the SNPs in Table 2 are false positive associations.

## Conclusions

Our results suggest that SNPs with similar case-control and family-based association results in designs that share probands are those that are most likely to replicate in independent populations, rather than the SNPs with the smallest p-values in either case-control or family-based designs alone. Thus, using probands from family-based studies in case-control designs, and combining results of both approaches, may be a way to augment our ability to find SNPs associated with asthma and other complex diseases.

## Competing interests

EKS received an honorarium for a talk on COPD genetics in 2006, and grant support and consulting fees from GlaxoSmithKline for two studies of COPD genetics. EKS received an honorarium from Bayer for a symposium at the ERS Meeting in 2005. EKS received honoraria in 2007 and 2008 and consulting fees from AstraZeneca. The remaining authors declare that there are no conflicts of interest to disclose.

## Authors' contributions

BEH participated in the design of the study, performed the statistical analysis and drafted the manuscript. ACW and BAR participated in the design of the study. BK performed genotyping experiments for CAMP and CR and helped draft the manuscript. JL, AJM, RL and CL performed the statistical analysis. JBW and GTO provided and analyzed data for the FHS population and helped draft the manuscript. GMH, MES, LA, and JCC provided data for the CR population and helped draft the manuscript. EKS, and STW participated in the design of the study and helped draft the manuscript. All authors read and approved the final manuscript.

## Pre-publication history

The pre-publication history for this paper can be accessed here:

http://www.biomedcentral.com/1471-2350/11/122/prepub

## Supplementary Material

Additional file 1 **Table S1**. CAMP/Illumina and CAMP Trio SNPs that replicated in CR with p-value < 0.05. Click here for file
