# Supplementary material for: Asthma-susceptibility variants identified using probands in case-control and family-based analyses
Source: BMC Med Genet. 2010 Aug 10;11:122. doi: 10.1186/1471-2350-11-122 (PMC2927535; doi:10.1186/1471-2350-11-122)
Supplement: Additional file 1 — Table S1. CAMP/Illumina and CAMP Trio SNPs that replicated in CR with p-value < 0.05. [file 1471-2350-11-122-S1.DOC]

**Table S1.** CAMP/Illumina and CAMP Trio SNPs that replicated in CR with p-value <0.05. Shown here are the 68 SNPs that did not pass Stage 2 (FHS or CAP p-value <0.05 and same direction of effect in FHS and CAP). The remaining 10 SNPs that had CR p-value <0.05 are in Table 2. CHR=chromosome; BP=base pair position along chromosome.

|  |  |  | p-value | | | | |
| --- | --- | --- | --- | --- | --- | --- | --- |
|  |  |  |  |  | Stage 1 | Stage 2 | |
| SNP | CHR | BP | CAMP/ Illumina | CAMP Trio | CR | FHS | CAP |
| rs6424099 | 1 | 24753713 | 8.0E-04 | 6.0E-05 | 0.038 | 0.93 | 3.8E-03 |
| rs16846809 | 1 | 172486021 | 6.7E-04 | 2.4E-03 | 0.034 | 0.32 | 0.56 |
| rs10489261 | 1 | 172707306 | 1.1E-04 | 5.6E-03 | 0.023 | 0.15 | 0.89 |
| rs6692753 | 1 | 173004080 | 4.2E-04 | 6.7E-03 | 0.023 | 0.33 | 0.93 |
| rs1562866 | 1 | 203523961 | 7.1E-03 | 7.3E-04 | 0.039 | 0.33 | 0.55 |
| rs2840967 | 1 | 223890651 | 3.7E-04 | 0.037 | 0.026 | 0.55 | 0.015 |
| rs3753214 | 1 | 239791545 | 7.9E-03 | 2.7E-02 | 2.2E-04 | 0.91 | 0.15 |
| rs12990732 | 2 | 54578537 | 3.5E-03 | 4.3E-03 | 0.045 | 0.85 | 0.61 |
| rs6734445 | 2 | 54651302 | 8.3E-04 | 9.8E-04 | 7.7E-03 | 0.78 | 0.53 |
| rs7584223 | 2 | 54672399 | 7.9E-04 | 6.4E-04 | 0.019 | 0.78 | 0.65 |
| rs6545435 | 2 | 54712907 | 3.5E-04 | 1.6E-04 | 0.037 | 0.86 | 0.74 |
| rs13033894 | 2 | 54863619 | 3.8E-03 | 2.4E-03 | 5.2E-03 | 0.57 | NA |
| rs1996634 | 2 | 76467666 | 7.6E-03 | 0.011 | 0.030 | 0.86 | 0.58 |
| rs830956 | 2 | 169820191 | 2.6E-03 | 1.7E-03 | 0.013 | NA | 0.57 |
| rs2241190 | 2 | 169823834 | 6.7E-03 | 7.7E-04 | 9.0E-03 | 0.84 | 0.48 |
| rs10498039 | 2 | 216034013 | 3.2E-04 | 0.044 | 0.036 | NA | 0.084 |
| rs4411688 | 2 | 216065067 | 1.1E-03 | 0.018 | 0.022 | 0.70 | 0.053 |
| rs7567258 | 2 | 216665174 | 5.4E-03 | 8.6E-03 | 0.019 | 0.69 | 0.23 |
| rs11706648 | 3 | 8771547 | 3.4E-03 | 0.017 | 6.6E-04 | 0.068 | 0.94 |
| rs2618406 | 3 | 12012909 | 3.1E-03 | 2.1E-03 | 0.028 | 0.37 | 0.54 |
| rs2618390 | 3 | 12022854 | 8.1E-03 | 0.033 | 0.018 | 0.15 | NA |
| rs10433430 | 3 | 141221335 | 2.5E-03 | 4.6E-03 | 0.041 | 0.20 | 0.77 |
| rs9844385 | 3 | 141222469 | 2.5E-03 | 4.6E-03 | 0.038 | 0.19 | 0.79 |
| rs1584586 | 3 | 151677041 | 5.8E-03 | 3.8E-03 | 6.3E-03 | 0.32 | 0.59 |
| rs11918915 | 3 | 183193967 | 6.6E-03 | 9.7E-03 | 0.022 | 0.64 | NA |
| rs4698414 | 4 | 15348071 | 1.3E-03 | 0.045 | 0.033 | 0.96 | 0.27 |
| rs1700687 | 5 | 76268760 | 3.1E-03 | 3.9E-04 | 0.034 | 0.33 | 0.41 |
| rs2055627 | 5 | 76270400 | 6.0E-03 | 0.014 | 0.019 | 0.24 | 0.45 |
| rs1875999 | 5 | 76300738 | 9.2E-03 | 2.1E-03 | 8.8E-04 | 0.060 | 0.51 |
| rs3863223 | 6 | 6281921 | 9.2E-03 | 1.1E-03 | 0.010 | 0.55 | 0.060 |
| rs6919094 | 6 | 10298044 | 5.2E-03 | 0.035 | 0.012 | 0.99 | NA |
| rs4712426 | 6 | 10304380 | 4.3E-03 | 0.028 | 0.011 | 1.00 | 0.38 |
| rs2502562 | 6 | 70651135 | 2.5E-03 | 0.031 | 0.026 | 0.41 | 0.56 |
| rs11751990* | 6 | 130829469 | 5.0E-06 | 0.037 | 0.018 | 0.81 | 0.43 |
| rs10245553 | 7 | 33100267 | 9.7E-03 | 0.050 | 0.013 | NA | 0.69 |
| rs2043787 | 7 | 38512024 | 2.5E-03 | 0.026 | 0.046 | 0.45 | 0.40 |
| rs7802385 | 7 | 41985224 | 6.6E-03 | 6.4E-04 | 0.018 | 0.69 | 0.16 |
| rs37070 | 7 | 81438483 | 1.6E-03 | 5.7E-03 | 0.046 | 0.73 | 0.79 |
| rs37071 | 7 | 81438724 | 1.4E-03 | 5.7E-03 | 0.049 | 0.73 | 0.88 |
| rs2106737 | 7 | 111365757 | 5.4E-03 | 0.019 | 3.8E-03 | 8.1E-03 | 1.00 |
| rs2410603 | 8 | 19056951 | 8.0E-03 | 0.046 | 0.045 | NA | 0.53 |
| rs1565623 | 8 | 143073127 | 8.8E-03 | 0.048 | 0.045 | 0.42 | NA |
| rs10733512 | 9 | 4354108 | 5.3E-03 | 0.048 | 0.017 | 0.50 | 0.34 |
| rs7854239 | 9 | 73467135 | 1.0E-03 | 5.2E-03 | 0.026 | 0.68 | 0.96 |
| rs10987080 | 9 | 127788222 | 4.1E-05 | 1.1E-04 | 2.6E-03 | 0.46 | NA |
| rs17268583 | 9 | 127818983 | 1.5E-03 | 0.050 | 0.036 | 0.25 | 0.70 |
| rs1220688 | 9 | 131361537 | 1.3E-03 | 0.017 | 0.026 | 0.070 | 0.41 |
| rs10828664 | 10 | 24854509 | 5.3E-03 | 0.013 | 0.015 | 0.87 | 0.11 |
| rs10764478 | 10 | 24892371 | 3.6E-03 | 0.024 | 7.3E-03 | 0.24 | 0.61 |
| rs10764142 | 10 | 38001713 | 9.4E-04 | 2.8E-03 | 0.015 | 0.85 | 0.76 |
| rs11011346 | 10 | 38049489 | 6.7E-04 | 7.7E-04 | 0.014 | 0.89 | 0.77 |
| rs624359 | 10 | 38339138 | 2.3E-03 | 2.3E-03 | 0.029 | 0.79 | 0.77 |
| rs7960991 | 12 | 45859650 | 2.8E-03 | 0.044 | 0.024 | 0.58 | 0.66 |
| rs10774816 | 12 | 114481876 | 9.7E-03 | 2.0E-03 | 0.012 | 0.82 | 0.58 |
| rs6575209 | 14 | 91214990 | 3.3E-03 | 0.029 | 9.3E-03 | 0.24 | 0.86 |
| rs8029386 | 15 | 64253490 | 9.7E-03 | 0.033 | 7.0E-03 | 0.17 | 0.94 |
| rs7164231 | 15 | 64270432 | 5.9E-03 | 0.041 | 7.8E-03 | 0.14 | NA |
| rs16949647 | 15 | 64276041 | 3.4E-03 | 0.044 | 0.027 | 0.056 | 0.62 |
| rs614342 | 18 | 27725315 | 5.6E-03 | 0.020 | 9.2E-03 | 0.28 | 0.11 |
| rs542237 | 18 | 63607085 | 8.5E-03 | 5.6E-03 | 0.013 | 0.66 | 0.28 |
| rs10405607 | 19 | 61376636 | 3.1E-04 | 0.029 | 9.0E-03 | 0.76 | 0.85 |
| rs761998 | 20 | 14257952 | 2.1E-03 | 0.029 | 0.011 | 0.11 | 0.31 |
| rs2837291 | 21 | 40207884 | 2.4E-03 | 3.1E-03 | 0.038 | 0.91 | 0.20 |
| rs2837295 | 21 | 40217036 | 3.7E-03 | 0.019 | 0.026 | 0.99 | 0.079 |
| rs465101 | 22 | 16903214 | 5.6E-03 | 0.026 | 0.028 | 0.55 | 0.55 |
| rs13056375 | 22 | 22082398 | 1.7E-03 | 4.5E-03 | 0.013 | 0.28 | 0.44 |
| rs9614891 | 22 | 43116310 | 1.5E-03 | 0.036 | 8.1E-03 | 0.24 | 0.41 |
| rs8137340 | 22 | 43120253 | 2.5E-05 | 6.4E-03 | 0.011 | 0.25 | 0.14 |
| *This SNP is also in Table 1. | | |  |  |  |  |  |
